# CrossMP: Enabling Cross-Modality Translation between Single-Cell RNA-Seq and Single-Cell ATAC-Seq through Web-Based Portal

**DOI:** 10.3390/genes15070882

**Published:** 2024-07-05

**Authors:** Zhen Lyu, Sabin Dahal, Shuai Zeng, Juexin Wang, Dong Xu, Trupti Joshi

**Affiliations:** 1 Department of Electrical Engineering and Computer Science, University of Missouri, Columbia, MO 65211, USA; zl7w2@missouri.edu (Z.L.); sddk7@missouri.edu (S.D.); zengs@missouri.edu (S.Z.); xudong@missouri.edu (D.X.); 2 Christopher S. Bond Life Sciences Center, University of Missouri, Columbia, MO 65211, USA; 3 Department of BioHealth Informatics, Luddy School of Informatics, Computing, and Engineering, Indiana University Indianapolis, Indianapolis, IN 46202, USA; wangjuex@iu.edu; 4 MU Institute for Data Science and Informatics, University of Missouri, Columbia, MO 65211, USA; 5 Department of Biomedical Informatics, Biostatistics and Medical Epidemiology, University of Missouri, Columbia, MO 65211, USA

**Keywords:** single-cell data analysis, scRNA-seq, scATAC-seq, co-assay, deep learning, cross-modality prediction

## Abstract

In recent years, there has been a growing interest in profiling multiomic modalities within individual cells simultaneously. One such example is integrating combined single-cell RNA sequencing (scRNA-seq) data and single-cell transposase-accessible chromatin sequencing (scATAC-seq) data. Integrated analysis of diverse modalities has helped researchers make more accurate predictions and gain a more comprehensive understanding than with single-modality analysis. However, generating such multimodal data is technically challenging and expensive, leading to limited availability of single-cell co-assay data. Here, we propose a model for cross-modal prediction between the transcriptome and chromatin profiles in single cells. Our model is based on a deep neural network architecture that learns the latent representations from the source modality and then predicts the target modality. It demonstrates reliable performance in accurately translating between these modalities across multiple paired human scATAC-seq and scRNA-seq datasets. Additionally, we developed CrossMP, a web-based portal allowing researchers to upload their single-cell modality data through an interactive web interface and predict the other type of modality data, using high-performance computing resources plugged at the backend.

## 1. Introduction

Single-cell measurements have revolutionized our understanding of cellular heterogeneity and diversity, allowing for the characterization of distinct cell types within complex tissues based on various molecular activities such as gene expression, chromatin accessibility, proteomics, and methylation. However, a significant constraint of current single-cell technologies is their capability to assess only one particular type of molecular activity per cell. For instance, a cell may undergo either single-cell RNA sequencing (scRNA-seq) or chromatin accessibility profiling (scATAC-seq), but not both. This restriction to a single molecular readout impedes our ability to comprehensively explore the interrelation of different genomic layers within individual cells [1] and understand the regulatory aspects.

Recent advancements in single-cell analysis have led to the emergence of multiomic single-cell methods, enabling the simultaneous profiling of multiple modalities within the same cell [2]. Unlike traditional approaches that focus solely on one omic data type in isolation, these multiomic methods facilitate integrated analysis across various molecular layers within individual cells. By adopting such holistic approaches, researchers can gain a deeper understanding of cellular behavior, elucidating how diverse omic layers, including gene expression, chromatin accessibility, DNA methylation, and protein expression, interact with and influence each other.

However, joint single-cell methods encounter various challenges apart from the technical limitations that can introduce errors or biases, further contributing to the noises in the resulting multiomic data [3]. Another significant obstacle is the increased cost associated with these multiomic experiments. The complexity and resource-intensive nature of performing such joint single-cell analyses can lead to higher expenses compared to traditional single-cell methods that focus on a single omic modality [2]. Additionally, the emergence of co-assays, in which multiple omic layers are simultaneously profiled from the same individual cells, is a more recent advancement in single-cell technology. Co-assay data is not as prevalent as single-assay data. Researchers may have limited access to co-assay datasets, and publicly available repositories might contain a smaller number of co-assay datasets compared to single-assay datasets. The existence of technical challenges and resource constraints makes it difficult to conduct joint profiling of multiple omic modalities within single cells.

Numerous methods have been developed to address challenges in single-cell data analysis. For scRNA-seq data, approaches such as SAUCIE [4], Deep Count Autoencoder [5], and scScope [6] have demonstrated efficacy in denoising data and capturing underlying biological variability. Similarly, for scATAC-seq data, models like cisTopic [7] and SCALE [8] have been successful in learning informative latent representations for clustering and regulatory region identification. Recent advancements in experimental techniques have facilitated the generation of paired single-cell data, enabling more efficient multimodal modeling approaches. For example, MultiVI [9] employs deep generative models to jointly analyze and integrate scRNA-seq and scATAC-seq data, leveraging variational autoencoders (VAEs) to embed both modalities into a shared latent space. Another notable model, BABEL [1], utilizes deep learning techniques to translate between gene expression and chromatin accessibility profiles at the single-cell level. However, there is still significant room for improvement in performance and accuracy. Additionally, the current models lack a user-friendly way to perform inference, which limits their accessibility and usability for a broader audience. Implementing pipelines, creating datasets, and transforming data to appropriately fit the model require users to be familiar with such processes and to invest significant time and effort. Furthermore, users need to access high-performance computing resources on Linux and learn how to run analyses in these environments. This can be a daunting task for those who are more accustomed to using less technical interfaces. By addressing these gaps, we can create a more efficient and user-centric solution.

In this paper, we propose a machine learning model, CrossMP, designed to computationally generate diverse multiomic modalities within a single cell from a solitary measured modality. The model is constructed using a deep neural network architecture, employing a fully connected deep network to learn the latent representation of each modality and predict the target modality. Our focus lies in bridging the gap between scRNA-seq and scATAC-seq profiles, enabling seamless translation between the two. Essentially, given an scRNA-seq profile of a set of cells, the model outputs the corresponding scATAC-seq profile, and vice versa. We trained our model using cells collected from various human and mouse datasets. Moreover, we integrated our pretrained model into the backend of a CrossMP web portal. This portal provides researchers with the capability to predict scRNA-seq and scATAC-seq data, offering a user-friendly platform for seamless access and utilization of our model’s predictive capabilities. The novelty of our approach lies in several aspects, including achieving superior accuracy performance compared to currently existing methods, providing a user-friendly web interface for users to conduct their own predictions, and actively developing capabilities for users to train models with their own datasets. These contributions aim to enhance accessibility and applicability in diverse research settings for a broader audience.

## 2. Materials and Methods

### 2.1. Data Preprocessing

The model was trained on a curated selection of paired human and mouse single-cell ATAC-seq and RNA-seq datasets sourced from the 10x Genomics multiomics platform (Table 1).

For the human subset, we compiled five distinct datasets. These include the COLO320DMHSR dataset, encompassing colon adenocarcinoma cells and colorectal adenocarcinoma cells. The kidney cancer dataset comprises human kidney nuclei obtained from frozen tissue. The lymphoma dataset features flash-frozen intra-abdominal lymph node tumor samples from a patient diagnosed with diffuse small lymphocytic lymphomas. Lastly, we have the PBMC I and PBMC II datasets. The former consists of peripheral blood mononuclear cells (PBMCs) from healthy male donors aged 30–35, whereas the latter comprises cryopreserved PBMCs from a healthy female donor aged 25.

For the mouse subset, we curated several datasets. The cortex dataset comprises 5081 and 10,309 nuclei from neonatal and adult mouse brains, respectively. The mouse brain dataset includes nuclei obtained from frozen brain tissue, while the mouse kidney dataset comprises nuclei extracted from frozen mouse kidney tissue. Additionally, we have the brain Alzheimer dataset, which involves a multiomic integration study con-ducted on a mouse model of Alzheimer’s disease.

**Figure 1:**
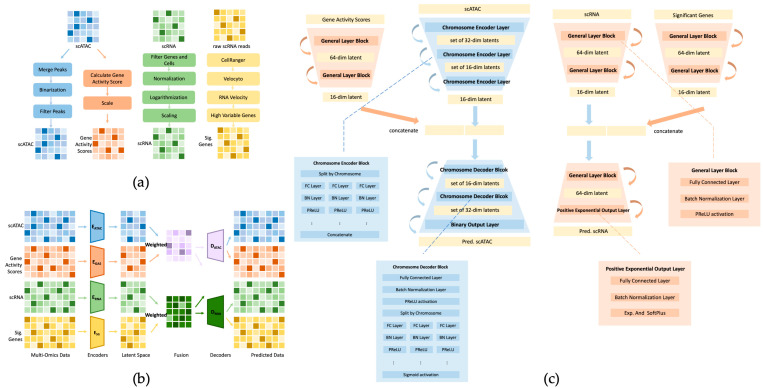
Overview of CrossMP model architecture. (**a**) CrossMP data pre-processing for each modality includes deriving the gene activity score from scATAC-seq and detecting significant genes through RNA velocity analysis. (**b**) Architecture of the CrossMP model. scRNA-seq, scATAC-seq, and two derived datasets—gene activity scores and significant gene expression—are used as inputs to four separate encoders. The latent representations of scATAC-seq and gene activity scores are then merged, as are those of scRNA-seq and significant gene expression. The two merged latent representations are subsequently processed through two decoders. Finally, we obtain four prediction results: ATAC to ATAC, ATAC to RNA, RNA to ATAC, and RNA to RNA. (**c**) The detailed structure of the CrossMP model includes a general encoder and decoder to process scRNA, gene activity scores, and significant gene modalities. Forward propagation is implemented through fully connected layers, a batch normalization layer, and PReLU activation. A specific encoder and decoder are used for the scATAC modality, enabling data to be split by chromosome and focus on intrachromosomal insights. The two 16-dimensional latent spaces from the same side are concatenated and fed into the decoders to obtain the predicted scATAC and scRNA.

To prepare the scATAC-seq data, several preprocessing steps were undertaken, as shown in Figure 1a. Initially, peaks located on sex chromosomes were excluded from consideration. Next, to streamline subsequent computation, overlapping peaks were merged into a unified representation. The resulting cell-by-peak matrix was binarized, with all nonzero values converted to 1, denoting the presence of chromatin accessibility, whereas absent regions were represented by 0. To foster the model’s capacity to discern generalizable patterns and features representative of the overall chromatin accessibility landscape, additional refinement steps were employed. Peaks that occurred infrequently, appearing in fewer than five cells, were eliminated to prevent overfitting to rare occurrences that may lack broad applicability across the dataset. Similarly, overly common peaks, observed in more than 10% of cells, were removed to mitigate potential biases toward highly prevalent regions that may not significantly contribute to distinguishing cell types.

Following the preprocessing steps applied to the scATAC-seq data, we prepared the scRNA-seq data similarly by removing the sex chromosomes. Subsequently, cells expressing fewer than 200 genes or more than 7000 genes were removed to ensure data quality and consistency. Subsequently, we standardized the data by adjusting the counts in each cell so that they totaled the median count per cell, ensuring uniformity of data across all cells. To address potential biases, we applied log transformation followed by Z-score normalization. More precisely, data points falling within the top and bottom 0.5% of the entire distribution were clipped. These normalization and filtering steps mitigated the influence of the extreme outliers, resulting in more reliable and balanced insights from our data. This approach enhanced the robustness and interpretability of our analysis.

Additionally, we derived gene activity scores using the regulatory potential (RP) model implemented within the MAESTRO suite [10], leveraging the scATAC-seq data. This model assessed the presence of scATAC-seq peaks surrounding each gene, indicating potential transcriptional regulator bindings and their impact on gene expression. Peaks were weighted by exponential decay from the transcription start site (TSS), and the sum of all peaks within a given gene exon region was calculated as if they were located at the TSS. This sum was then normalized by the total exon length. By inputting our scATAC-seq data into the RP model, we obtained the gene activity score corresponding to the scATAC-seq data with a 10 k decay distance using the enhanced model.

Furthermore, we acquired the raw FASTQ sequences of the scRNA-seq data and subsequently processed these raw sequence files using 10x Genomics CellRanger 3.1.0 [11] to generate raw feature-barcode matrices, along with the intermediate BAM file. This BAM file was then used with Velocyto [12] to convert it to the LOOM format, facilitating downstream analysis. Finally, utilizing scVelo [13] with the LOOM file as input, we identified significant genes ranked by the velocity score.

### 2.2. Model Architecture

The model consists of four encoder networks and two decoder networks. Each encoder independently projects the scRNA-seq, scATAC-seq, gene activity scores, and significant gene expression into the latent space. At the bottleneck layer, we merged the latent representation from scRNA-seq and significant gene expression by using element-wise addition. Likewise, the latent representation derived from scATAC-seq and the gene activity scores were merged. The decoders were then utilized to infer the scRNA-seq and scATAC-seq outputs from the latent representation (Figure 1b).

As shown in Figure 1c, in the encoders for scRNA-seq and significant genes, we initially projected the gene–cell expression matrix into a 16-dimensional latent space through two fully connected layers (FC layers), each followed by batch normalization layer (BN layers) and ReLU (rectified linear unit) activation. Subsequently, we performed an element-wise merge of the two resulting latent representations. In the decoder for scRNA-seq, the 16-dimensional latent space was first expanded to a 64-dimensional space. Then, this 64-dimensional space was further processed to produce two outputs of the same dimensionality as the input. Finally, these outputs underwent exponential activation functions to calculate the mean and softplus activation and the dispersion parameters.

In the encoders for scATAC-seq and derived gene activity scores, rather than simply projecting the genome-wide peak information with a single fully connected layer, we split the whole-genome peaks by chromosome and assigned a fully connected network to process the peaks of each chromosome independently. This strategy aimed to shed light on the intrachromosomal interaction of DNA accessibility rather than focusing solely on interactions across different chromosomes. Every fully connected network contained two fully connected layers to project the input onto a 16-dimensional space, each followed by a PReLU (parametric ReLU) activation. Subsequently, we concatenated all the resulting latent representations to yield a 352-dimensional concatenated representation. This concatenated representation was then projected onto a 16-dimensional latent representation with the PReLU activation. Following this, we performed an element-wise merge of the 16-dimensional latent representations from scATAC-seq and gene activity scores. Moving to the decoder for scATAC-seq, we began by projecting the 16-dimensional latent representation onto a 352-dimensional space using the PReLU activation. This representation was then split into 22 blocks, each representing a chromosome, with each block containing a 16-dimensional space. We assigned a separate fully connected network to each latent representation, restoring the dimensions to their original sizes for each chromosome, followed by applying a sigmoid activation function.

MP model was implemented based on Python version 3.8.16, Pytorch version 1.13.1, cpuonly version 2.0, Skorch version 0.11.0, Anndata version 0.8.0, Scanpy version 1.9.1, Matplotlib vrsion 3.6.3, Pandas version 1.5.3, Scikit-learn version 1.2.0, R version 4.0.5, MAESTRO version 1.5.1, CellRanger version 3.1.0, Velocyto version 0.17.17, scVelo version 0.2.5.

### 2.3. Model Training

The ATAC encoder, gene activity score encoder, RNA encoder, and significant gene encoder are denoted as $E_{ATAC}$, $E_{GAS}$, $E_{RNA},\;\mathrm{and}E_{SG}$, respectively. These encoders construct the low-dimensional embeddings $X_{ATAC}^{embed}$, $X_{GAS}^{embed}$, $X_{RNA}^{embed}$, and $X_{SG}^{embed}$ from the input scATAC ($X_{ATAC}$), gene activity scores ($X_{GAS}$), scRNA ($X_{RNA}$), and significant genes ($X_{SG}$), as shown in Equation (1).

(1) $$X_{ATAC}^{embed}=E_{ATAC}\left(X_{ATAC}\right)\;X_{GAS}^{embed}=E_{GAS}\left(X_{GAS}\right)\;X_{RNA}^{embed}={\;E}_{RNA}\left(X_{RNA}\right)\;X_{SG}^{embed}={\;E}_{SG}\left(X_{SG}\right)$$

In the bottleneck layer, we concatenated the embeddings ($X_{ATAC}^{embed}$, $X_{GAS}^{embed}$) and ($X_{RNA}^{embed}$, $X_{SG}^{embed}$).

(2) $$X_{ATAC+GAS}^{embed}=Concatenate\left(X_{ATAC}^{embed}+X_{GAS}^{embed}\right)\;X_{RNA+SG}^{embed}=Concatenate\left(X_{RNA}^{embed}+X_{SG}^{embed}\right)$$

The ATAC decoder and RNA decoder are denoted as $D_{ATAC}$ and $D_{RNA}$ for reconstructing the scATAC and scRNA, respectively. Here, $X_{ATAC\to ATAC}^{pred}$ represents the reconstructed scATAC from scATAC, $X_{ATAC\to RNA}^{pred}$ represents the predicted scRNA from scATAC, $X_{RNA\to ATAC}^{pred}$ represents the predicted scATAC from scRNA, and $X_{RNA\to RNA}^{pred}$ represents the reconstructed scRNA from scRNA.

(3) $$X_{ATAC\to ATAC}^{pred}=D_{ATAC}\left(X_{ATAC+GAS}^{embed}\right)\;X_{ATAC\to RNA}^{pred}=D_{RNA}\left(X_{ATAC+GAS}^{embed}\right)\;X_{RNA\to ATAC}^{pred}={\;D}_{ATAC}\left(X_{RNA+SG}^{embed}\right)\;X_{RNA\to RNA}^{pred}={\;D}_{RNA}\left(X_{RNA+SG}^{embed}\right)$$

To assess the accuracy of the inferred scRNA-seq data, whether generated from scRNA-seq or scATAC-seq, we employed the negative binomial (NB) loss function, denoted as $L_{NB}$. This choice was informed by its efficacy, proven in previous studies, in terms of imputing and denoising single-cell expression data [5,14]. Similarly, to gauge the accuracy of the inferred scATAC-seq data, whether generated from scRNA-seq or scATAC-seq, we utilized the binary cross-entropy (BCE) loss function, denoted as $L_{BCE}$. This loss function is well-suited to evaluating binary predictions, making it a natural choice for deep learning models applied to scATAC-seq data. Additionally, we computed the KL (Kullback–Leibler) divergence loss, denoted as $L_{KL}$, between the two bottleneck latent representations to further evaluate the similarity between the two latent representations. Finally, we derived the loss function as follows:

(4) $$\begin{array}{cc} Loss= & L_{NB}\left(X_{ATAC\to ATAC}^{pred},\;X_{ATAC}\right)+\omega_{BCE}\left(L_{BCE}\left(X_{ATAC\to RNA}^{pred},\;X_{RNA}\right)\right)\\ & +L_{NB}\left(X_{RNA\to ATAC}^{pred},\;X_{A}\right)+\omega_{BCE}\left(L_{BCE}\left(X_{RNA\to RNA}^{pred},\;X_{RNA}\right)\right)\\ & +\omega_{KL}\left(L_{KL}\left(X_{ATAC+GAS}^{embed},X_{RNA+SG}^{embed}\right)\right) \end{array}$$

We trained the model using the Adam optimizer with a learning rate of 0.01. Early stopping was set to 25 epochs. The batch size was 512 during training. We set $\varphi_{BCE}$ = 1.33 and $\varphi_{KL}$= 1 for all the training datasets.

### 2.4. Web Server Implementation

**Figure 2:**
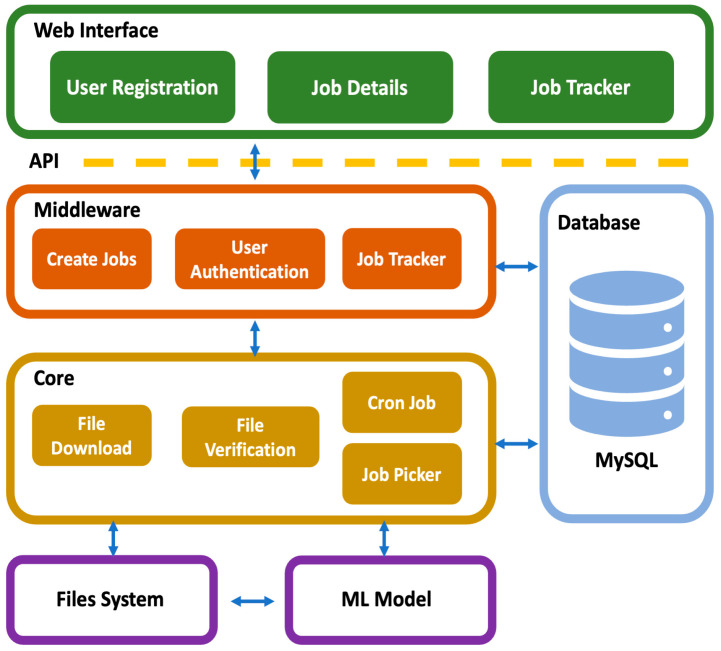
Architecture of the CrossMP web portal. The architecture consists of four modules, which communicate with each other via appropriate APIs.

For easier access to the developed models and results, a web-based portal, CrossMP, was developed with a lightweight development environment and hosted on Docker [15]. Designed to enhance user experience, the system offers clean and well-organized interface components, which help to minimize operational errors. By leveraging high-performance computing resources, it ensures efficient, sustainable, and reliable performance even under heavy workloads. CrossMP generates unique user identifiers to store all input files, models, and result files securely, maintaining privacy and confidentiality. The CrossMP architecture is structured into four distinct modules (Figure 2).

#### 2.4.1. Web Interface Module

This module utilizes lightweight UI libraries like AngularJS [16] to ensure user-friendliness. Its responsive design ensures a consistent appearance across various screen sizes, whether on a computer or a tablet. Additionally, it is compatible with multiple cross-platform web browsers, including Google Chrome, Firefox, Microsoft Edge, and Safari.

#### 2.4.2. Middleware Module

This module serves as an intermediary between the web interface and the database. It employs a RESTful API built with PHP, which leverages HTTP requests for data access and retrieval, job creation, and job information display. To ensure security, a token-based login system and token-based authentication validate each API request. The API interacts with AngularJS on the front end.

#### 2.4.3. Core Module

The core modules mainly consist of the file download, file verification, and a job picker that can run synchronously from the main application. The file download module is called whenever the job is created, and it uses Google API to access the file from Google Drive and the stream download method because the file is likely to be large. The job picker module is called by the cronjob that runs periodically, and it checks the available core and running job to determine which jobs can be run to properly utilize the hardware resources without overloading them. It also uses Python data analysis libraries, such as Scanpy [17] and Pandas, to validate uploaded files. This module is also responsible for sending notifications to the user about the successful and failed jobs.

#### 2.4.4. Database Module

MySQL [18] databases are used in this module. Taking advantage of a relational database, they help keep track of the user data and the statuses of not started, running, failed, and successful jobs.

## 3. Results

### 3.1. Evaluation and Metrics

To demonstrate the performance of predictions of scRNA-seq and scATAC-seq, we compared the model with the previously mentioned BABEL model and scButterfly [19]. We implemented BABEL using its respective GitHub repository with default parameters. For scButterfly, we adapted the scButterfly-B model because cell types were not available during training, and we used the same feature selection strategy as CrossMP for comparable results. We randomly split all datasets, assigning 70% of cells to the training set, 15% to the validation set, and 15% to the test set. The performance of the scRNA-seq data using the Pearson and Spearman correlation coefficient and the scATAC-seq data was evaluated using the area under the receiver operating characteristic (AUROC) curve.

CrossMP achieved strong performance for cross-modality inference. Inferring RNA expression from ATAC accessibility on the human COLO320DMHSR dataset, it achieved a Pearson correlation of 0.680 and a Spearman’s correlation of 0.616 (Table 2). Inferring ATAC from RNA on the human lymphoma dataset, CrossMP achieved an AUROC of 0.861. Its performance extended to mouse datasets as well. On the mouse kidney dataset, it achieved a Pearson correlation of 0.530 and a Spearman’s correlation of 0.404. Additionally, on the mouse cortex dataset, CrossMP achieved an AUROC of 0.890 (Table 3).

**Figure 3:**
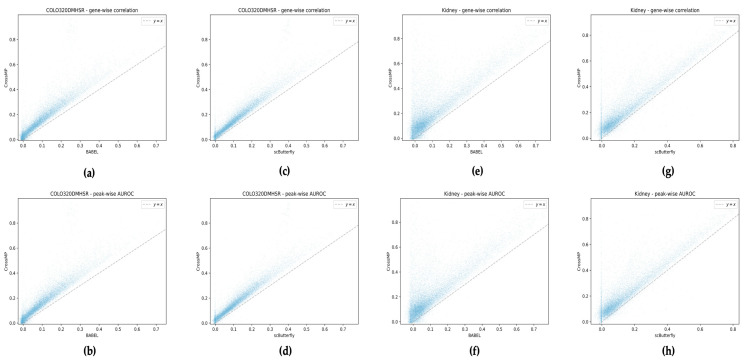
CrossMP gene-wise and peak-wise correlation compared with BABEL and scButterfly. (**a**) Gene-wise correlation and (**b**) peak-wise AUROC between the ground truth and predicted result of the COLO320DMHSR human dataset, comparing CrossMP with BABEL; (**c**) gene-wise correlation and (**d**) peak-wise AUROC between the ground truth and predicted result of the COLO320DMHSR human dataset, comparing CrossMP with scButterfly; (**e**) gene-wise correlation and (**f**) peak-wise AUROC between the ground truth and predicted result of the mouse kidney dataset, comparing CrossMP with BABEL; (**g**) gene-wise correlation and (**h**) peak-wise AUROC between the ground truth and predicted result of the mouse kidney dataset, comparing CrossMP with scButterfly.

To evaluate the performance of our model, we also measured how well the predicted RNA and ATAC profiles allowed us to recapitulate gene expression and peak differences across cells. To achieve this, we calculated the gene-wise correlation and peak-wise AUROC between the predicted profile and the true normalized profile [20]. In this analysis, CrossMP demonstrated superior performance compared to BABEL and scButterfly. Notably, CrossMP significantly outperformed BABEL and the scButterfly model trained on the human COLO320DMHSR dataset and mouse kidney dataset (Figure 3). Furthermore, CrossMP’s performance remained consistent across various human and mouse datasets (Supplementary Materials, Figures S1–S4).

### 3.2. CrossMP Web Portal and Job Submission

**Figure 4:**
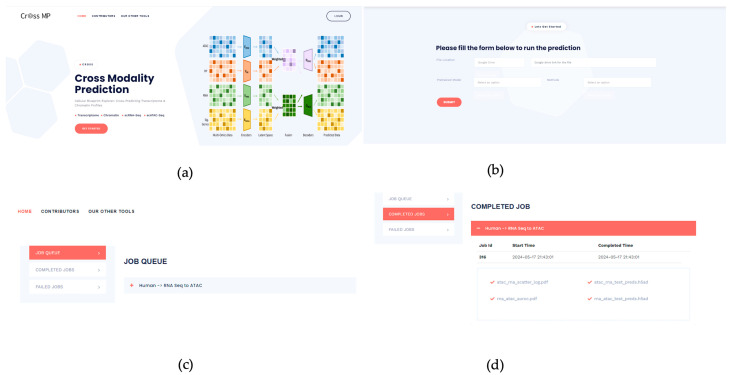
CrossMP job creation and results verification. (**a**) Home page of CrossMP web portal. (**b**) CrossMP job submission form. (**c**) Job tracker: monitoring ongoing, failed, and completed jobs. (**d**) Completed job section: view and download results.

CrossMP is publicly available at https://crossmp.missouri.edu (accessed on 2 July 2024). Clicking “Get Started” in Figure 4a will take users to the registration page if they have not already done so. After registering and signing in, users can navigate to the interface shown in Figure 4b to create a job. Users can choose the file location option, either Google Drive or a direct download link. If one selects Google Drive, they can create a shareable link with the access level set to “anyone with the link can access”, then paste it into the input field. The input file should be in h5ad format and contain the scRNA-seq or scATAC-seq data. Next, users can select the pretrained model by clicking the “Pretrained model” dropdown list. Then, users need to choose the prediction direction using the “Method” dropdown list to specify whether it is from scATAC-seq to scRNA-seq or vice versa. Finally, after clicking “Submit”, the job will run in the background. Notifications will be sent if the job fails or completes. Meanwhile, users can click on their name in the top-right corner to open job trackers. This section will display all queued, completed, and failed jobs, as shown in Figure 4c. Users can access comprehensive job results by navigating to the “Completed Jobs” section and clicking the collapse symbol next to each job. This action reveals the predicted results, including a clustering UMAP visualization, contained within the associated h5ad file, as shown in Figure 4d.

## 4. Conclusions

We introduced a machine learning model designed to effectively bridge the gap between scRNA-seq and scATAc-seq profiles using co-assay single-cell data. Through the comprehensive evaluation, we have demonstrated the robust performance of our model across diverse experimental contexts, including holdout test datasets and those generated using different experimental protocols. This underscores its versatility and robustness in accurately translating between modalities, thereby facilitating comprehensive analysis of single-cell omics data. Furthermore, we also thoroughly examined the potential limitations of CrossMP. Firstly, it tends to achieve superior results with large datasets, whereas its performance diminishes with smaller datasets comprising fewer than 10,000 cells. This suggests that CrossMP performs sub-optimally with smaller datasets (Supplementary Materials, Table S1), which we plan to investigate further.

In addition to its performance, our model is accompanied by the user-friendly CrossMP web portal. This portal boasts an intuitive and interactive interface, empowering researchers to effortlessly harness the predictive capabilities of our model. By simply uploading their input modality data file into the specific h5ad format, researchers can seamlessly predict scRNA-seq or scATAC-seq data. Moreover, the portal offers advanced functionalities and visualization tools to further streamline data analysis and interpretation, fostering collaboration and accelerating discoveries in the field of single-cell omics.

In our future endeavors, we aim to enhance the performance of our pretrained human model by augmenting our dataset with additional human co-assay data. By expanding our dataset, we can improve the model’s accuracy and generalizability, enabling more robust translation of single-cell omics data. Furthermore, we intend to enhance our model’s capabilities by expanding its translation abilities to encompass a variety of organisms, including plants such as soybean, maize, Arabidopsis, and other species. This expansion will broaden the applicability of our model and facilitate cross-species comparisons in single-cell omics research. Additionally, we aspire to extend our model to accommodate translation between other single-cell modalities, such as single-cell proteomics data, in the future.

In parallel, we seek to enhance the functionality of the CrossMP web portal to empower users to train their own models using their own datasets. This feature will enable researchers to tailor the model to their specific experimental setups and biological questions, fostering customization and flexibility in single-cell omics analysis.

## Figures and Tables

**Table 1:** Dataset summary.

Organism	Dataset	Cells	Genes	Peaks
Human	COLO320DMHSR	~71 k	~19 k	~333 k
	Kidney Cancer	~22 k	~34 k	~49 k
	Lymphoma	~14 k	~34 k	~92 k
	PBMC I	~10 k	~34 k	~85 k
	PBMC II	~11 k	~34 k	~117 k
Mouse	Cortex	~15 k	~17 k	~285 k
	Brain	~23 k	~30 k	~70 k
	Kidney	~14 k	~30 k	~53 k
	Brain Alzheimer	~33 k	~30 k	~57 k

**Table 2:** CrossMP performance was evaluated on five human datasets. The performance of ATAC-to-RNA translation was assessed using Pearson and Spearman correlation coefficients, whereas RNA-to-ATAC translation was evaluated using AUROC. We compared the performance of CrossMP with that of BABEL and scButterfly.

Dataset	Evaluation Metric	CrossMP	BABEL	scButterfly
COLO320DMHSR	Pearson’s r	**0.680**	0.663	0.671
	Spearman’s r	0.616	0.609	**0.619**
	AUROC	**0.817**	0.765	0.777
Kidney Cancer	Pearson’s r	**0.459**	0.427	0.449
	Spearman’s r	0.352	0.348	**0.362**
	AUROC	**0.821**	0.704	0.654
Lymphoma	Pearson’s r	0.390	0.376	**0.393**
	Spearman’s r	0.290	0.291	**0.336**
	AUROC	**0.861**	0.808	0.524
PBMC I	Pearson’s r	0.362	0.426	**0.435**
	Spearman’s r	0.293	0.294	**0.332**
	AUROC	**0.852**	0.815	0.765
PBMC II	Pearson’s r	**0.495**	0.475	0.490
	Spearman’s r	0.344	0.342	**0.389**
	AUROC	**0.856**	0.824	0.534

**Table 3:** CrossMP performance was evaluated on five mouse datasets. The performance of ATAC-to-RNA translation was assessed using Pearson and Spearman correlation coefficients, whereas RNA-to-ATAC translation was evaluated using AUROC. We compared the performance of CrossMP with that of BABEL and scButterfly.

Dataset	Evaluation Metric	CrossMP	BABEL	scButterfly
Cortex	Pearson’s r	0.300	0.290	**0.302**
	Spearman’s r	0.244	0.247	**0.251**
	AUROC	**0.890**	0.861	0.856
Brain	Pearson’s r	**0.511**	0.485	0.500
	Spearman’s r	0.414	0.409	**0.439**
	AUROC	**0.836**	0.751	0.570
Kidney	Pearson’s r	**0.530**	0.488	0.511
	Spearman’s r	0.404	0.397	**0.441**
	AUROC	**0.849**	0.767	0.775
Brain Alzheimer	Pearson’s r	**0.522**	0.486	0.511
	Spearman’s r	0.413	0.407	**0.433**
	AUROC	**0.816**	0.722	0.519

## Data Availability

Single-cell multiome ATAC + gene expression profiling of the COLO320-DM and COLO320-HSR cell lines data are available through Gene Expression Omnibus (GEO; accession no. GSE160148). Human kidney nuclei data from frozen tissue are accessible through the 10x Genomics data portal (https://www.10xgenomics.com/datasets/human-kidney-cancer-nuclei-isolated-with-chromium-nuclei-isolation-kit-saltyez-protocol-and-10x-complex-tissue-dp-ct-sorted-and-ct-unsorted-1-standard). Data on flash-frozen intra-abdominal lymph node tumors from patients diagnosed with diffuse small lymphocytic lymphoma are accessible through the 10x Genomics data portal (https://www.10xgenomics.com/datasets/fresh-frozen-lymph-node-with-b-cell-lymphoma-14-k-sorted-nuclei-1-standard-2-0-0). Data on peripheral blood mononuclear cells (PBMCs) from a healthy male donor aged 30–35 are available through the 10x Genomics data portal (https://www.10xgenomics.com/datasets/10-k-human-pbm-cs-multiome-v-1-0-chromium-x-1-standard-2-0-0). Data on cryopreserved peripheral blood mononuclear cells (PBMCs) from a healthy female donor aged 25 are available through the 10x Genomics data portal (https://www.10xgenomics.com/datasets/pbmc-from-a-healthy-donor-granulocytes-removed-through-cell-sorting-10-k-1-standard-2-0-0) The mouse brain cortex dataset is available through GEO; accession no. GSE126074). Nuclei from frozen mouse brain tissue are available through the 10x Genomics data portal. (https://www.10xgenomics.com/datasets/mouse-brain-nuclei-isolated-with-chromium-nuclei-isolation-kit-saltyez-protocol-and-10x-complex-tissue-dp-ct-sorted-and-ct-unsorted-1-standard). Nuclei from frozen mouse kidney tissue are available through the 10x Genomics data portal (https://www.10xgenomics.com/datasets/mouse-kidney-nuclei-isolated-with-chromium-nuclei-isolation-kit-saltyez-protocol-and-10x-complex-tissue-dp-ct-sorted-and-ct-unsorted-1-standard). Data on single cell multiome RNA + ATAC from an Alzheimer’s Disease Mouse Model Brain is available through the 10x Genomics data portal (https://www.10xgenomics.com/datasets/multiomic-integration-neuroscience-application-note-single-cell-multiome-rna-atac-alzheimers-disease-mouse-model-brain-coronal-sections-from-one-hemisphere-over-a-time-course-1-standard). CrossMP is available as a web-based portal at https://crossmp.missouri.edu. The Python-based deep-learning model is available in the GitHub repository (https://github.com/tang27abu/CrossMP).

## Supplementary Materials

The following supporting information can be downloaded at: https://www.mdpi.com/article/10.3390/genes15070882/s1, Figure S1. CrossMP gene-wise/peak-wise correlation compared with BABEL. (a,b) Gene-wise correlation and peak-wise AUROC between the ground truth and predicted result of human kidney cancer dataset, comparing CrossMP with BABEL; (c,d) comparing CrossMP with BABEL on human lymphoma dataset; (e,f) comparing CrossMP with BABEL on human PBMC I dataset; (g,h) comparing CrossMP with BABEL on human PBMC II dataset. Figure S2. CrossMP gene-wise/peak-wise correlation compared with BABEL. (a,b) Gene-wise correlation and peak-wise AUROC between the ground truth and predicted result of mouse brain cortex, comparing CrossMP with BABEL; (c,d) comparing CrossMP with BABEL on mouse brain dataset; (e,f) comparing CrossMP with BABEL on mouse brain Alzheimer dataset. Figure S3. CrossMP gene-wise/peak-wise correlation compared with scButterfly. (a,b) Gene-wise correlation and peak-wise AUROC between the ground truth and predicted result of human lymphoma dataset, comparing CrossMP with scButterfly; (c,d) comparing CrossMP with scButterfly on human PBMC I dataset; (e,f) comparing CrossMP with scButterfly on human PBMC II dataset. Figure S4. CrossMP gene-wise/peak-wise correlation compared with scButterfly. (a,b) Gene-wise correlation and peak-wise AUROC between the ground truth and predicted result of mouse brain cortex, comparing CrossMP with scButterfly; (c,d) comparing CrossMP with scButterfly on mouse brain dataset; (e,f) comparing CrossMP with scButterfly on mouse brain Alzheimer dataset. Table S1. Datasets summary and performance information.
