# Supplementary material for: CrossMP: Enabling Cross-Modality Translation between Single-Cell RNA-Seq and Single-Cell ATAC-Seq through Web-Based Portal
Source: Genes (Basel). 2024 Jul 5;15(7):882. doi: 10.3390/genes15070882 (PMC11276538; doi:10.3390/genes15070882)
Supplement: Supplementary file 1 [file genes-15-00882-s001.zip › genes-3051002-supplementary.pdf]

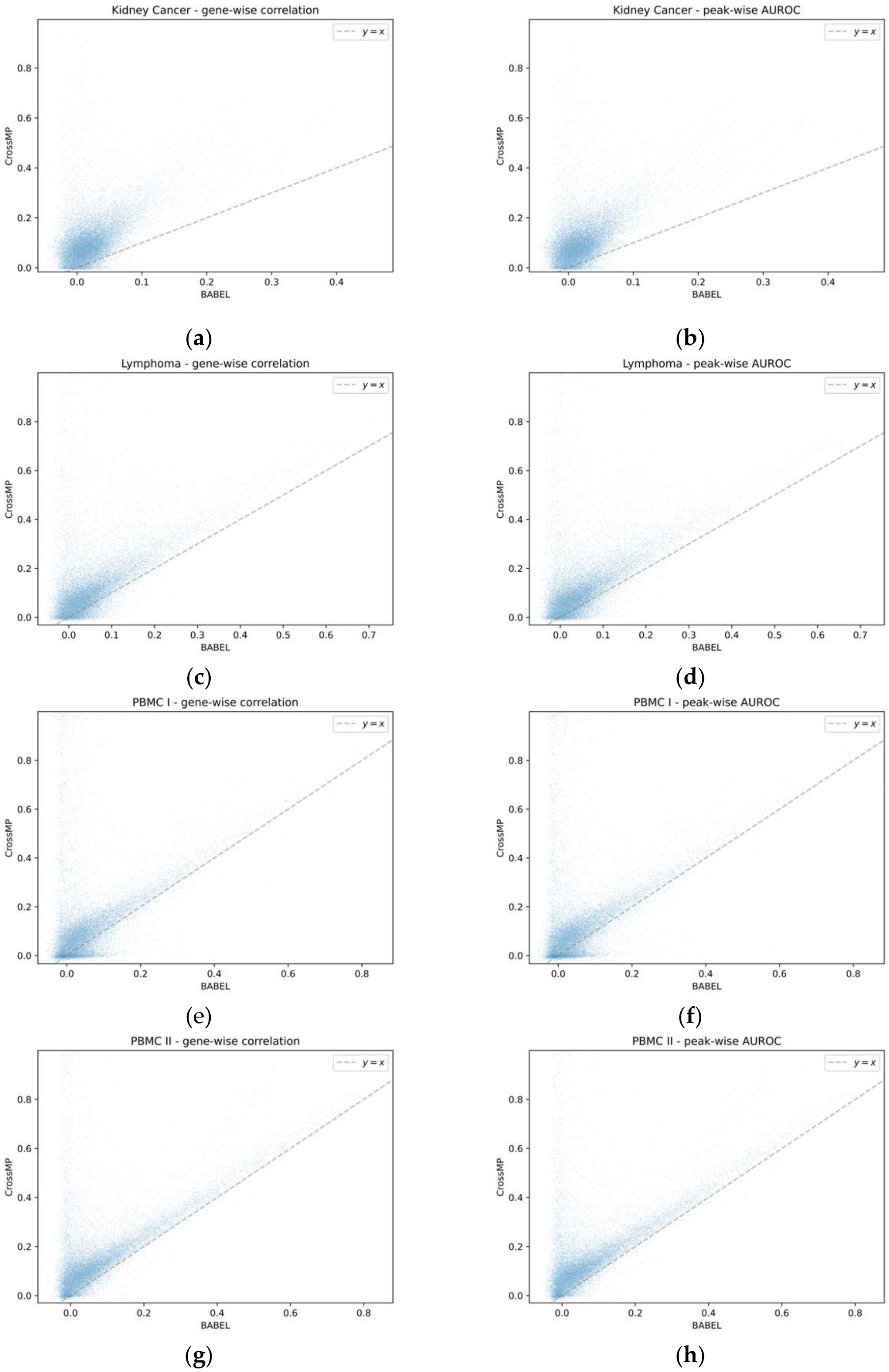

Figure S1. CrossMP gene-wise/peak-wise correlation compared with BABEL**.** (**a**)(**b**) Gene-wise correlation and peak-wise AUROC between the ground truth and predicted result of human kidney cancer dataset, comparing CrossMP with BABEL; (**c**) (**d**) comparing CrossMP with BABEL on human lymphoma dataset; (**e**) (**f**) comparing CrossMP with BABEL on human PBMC I dataset; (**g**) (**h**) comparing CrossMP with BABEL on human PBMC II dataset.

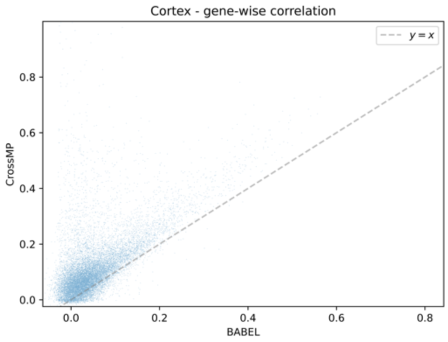

(**a**)

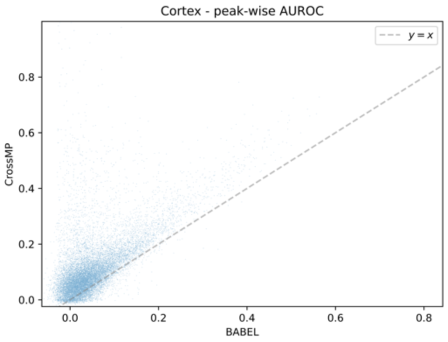

(**b**)

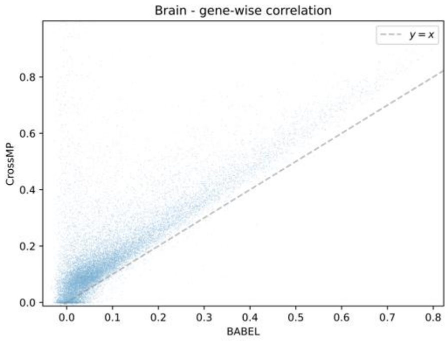

(**c**)

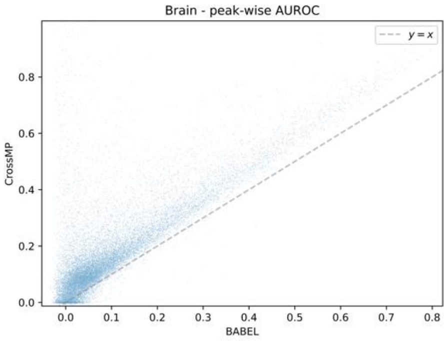

(**d**)

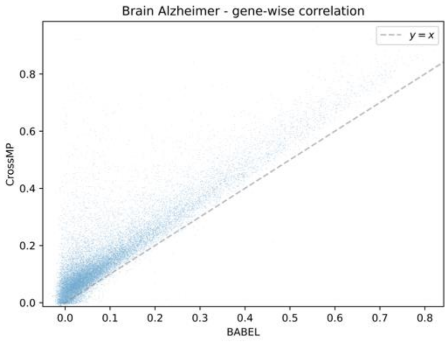

(**e**)

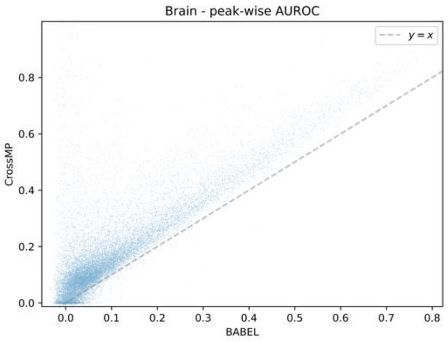

(**f**)

Figure S2. CrossMP gene-wise/peak-wise correlation compared with BABEL. (**a**)(**b**) Gene-wise correlation and peak-wise AUROC between the ground truth and predicted result of mouse brain cortex, comparing CrossMP with BABEL; (**c**) (**d**) comparing CrossMP with BABEL on mouse brain dataset; (**e**) (**f**) comparing CrossMP with BABEL on mouse brain Alzheimer dataset.

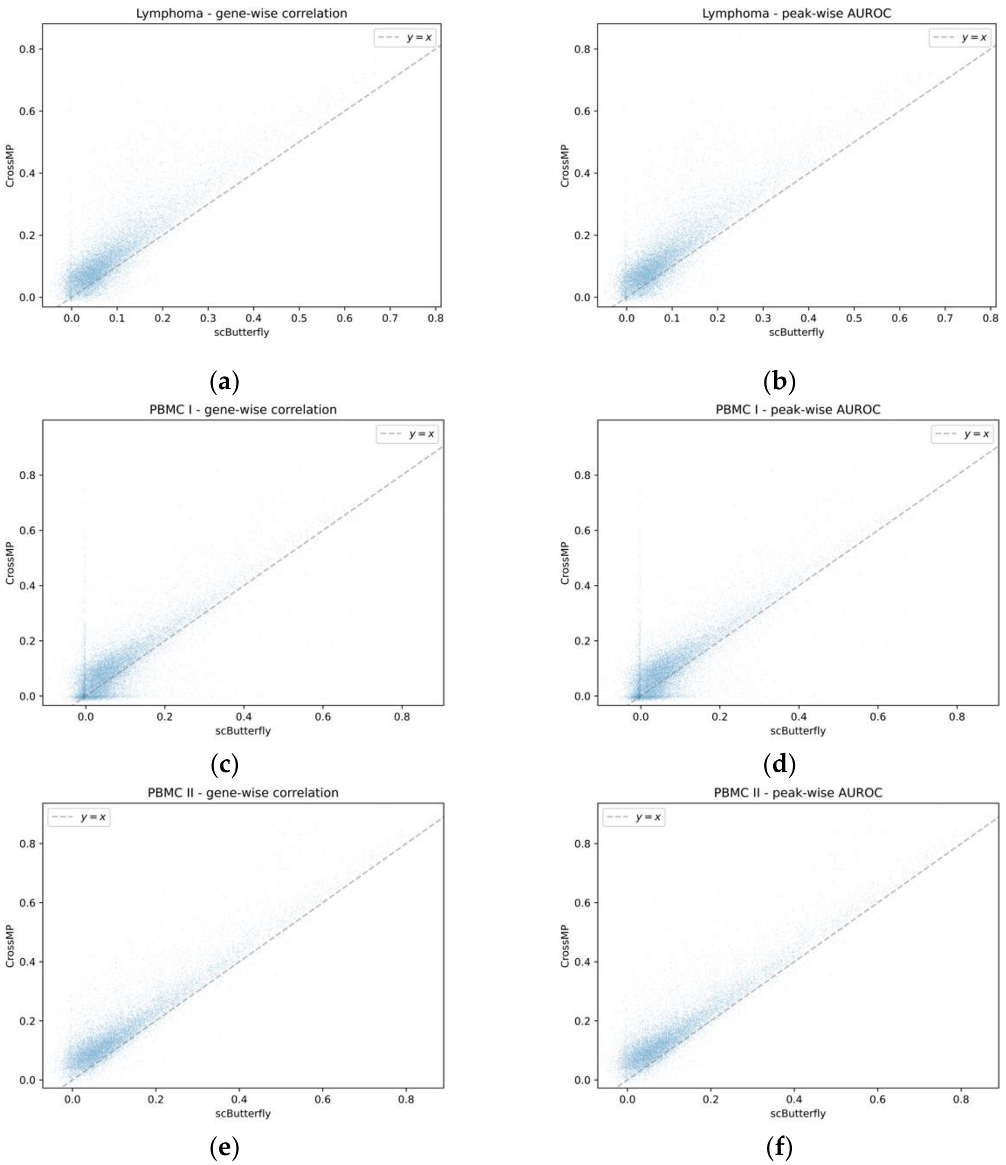

Figure S3. CrossMP gene-wise/peak-wise correlation compared with scButterfly. (**a**)(**b**) Gene-wise correlation and peak-wise AUROC between the ground truth and predicted result of human lymphoma dataset, comparing CrossMP with scButterfly; (**c**) (**d**) comparing CrossMP with scButterfly on human PBMC I dataset; (**e**) (**f**) comparing CrossMP with scButterfly on human PBMC II dataset.

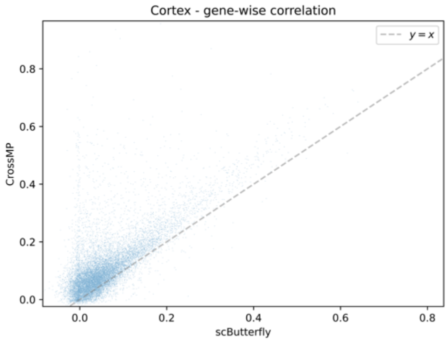

(**a**)

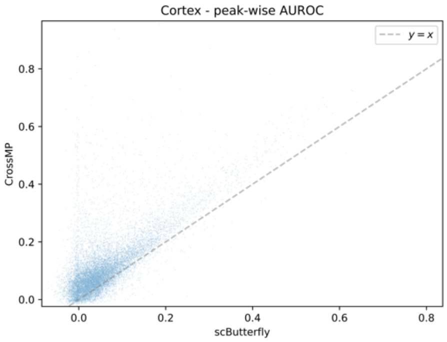

(**b**)

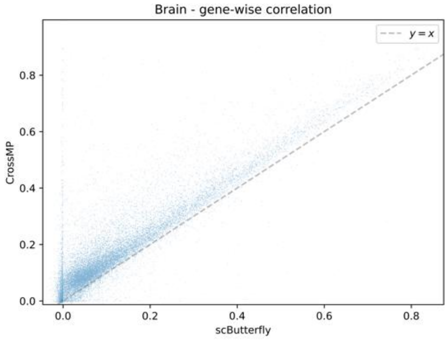

(**c**)

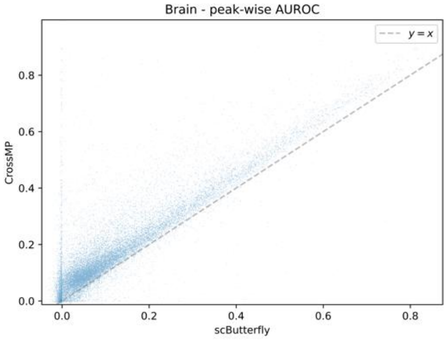

(**d**)

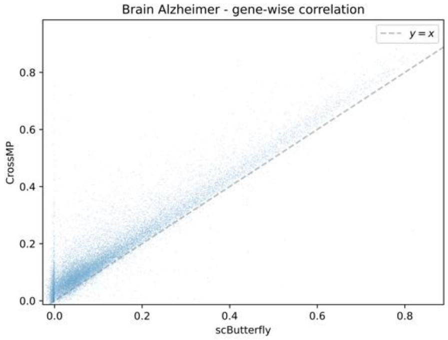

(**e**)

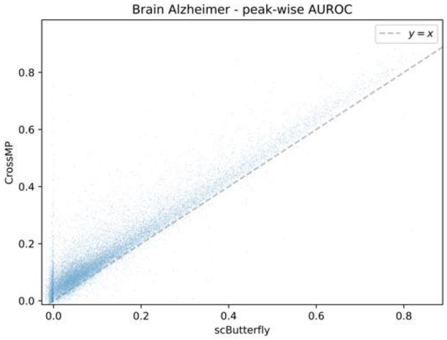

(**f**)

Figure S4. CrossMP gene-wise/peak-wise correlation compared with scButterfly. (**a**)(**b**) Gene-wise correlation and peak-wise AUROC between the ground truth and predicted result of mouse brain cortex, comparing CrossMP with scButterfly; (**c**) (**d**) comparing CrossMP with scButterfly on mouse brain dataset; (**e**) (**f**) comparing CrossMP with scButterfly on mouse brain Alzheimer dataset.

Table S1. Datasets summary and performance information.

Organism	Dataset	Cell Type	Cells	Pearson's r	Spearman's r	AUROC
Human	COLO320 DMHSR	colon adenocarcinoma/ colorectal adenocarcinoma	~71k	0.680	0.616	0.817
	**Brain**	**brain**	**~7k**	**0.146**	**0.340**	**0.860**
	PBMC II	blood	~11k	0.495	0.344	0.856
Mouse	**E18 Brain**	**brain**	**~4k**	**0.470**	**0.439**	**0.838**
	Kidney	kidney	~14k	0.530	0.404	0.849

This table summarizes CrossMP performance on three human and two mouse datasets, along with relevant dataset information. CrossMP performance is typically sub-optimal for datasets with low number of cells (< ~10k) such as on the human brain (7k cells) and mouse E18 brain (4k cells) datasets, which are shown in bold.
